# Changes in parental knowledge and concerns regarding pediatric fever from 2017 to 2024: repeated cross-sectional surveys on the association of a smartphone application

**DOI:** 10.3389/fpubh.2025.1619134

**Published:** 2026-01-06

**Authors:** Masahiko Sakamoto, Asuka Suzuki, Hirono Ishikawa

**Affiliations:** 1 Department of Pediatrics, Saku Central Hospital Advanced Care Center, Saku, Japan; 2 Teikyo University Graduate School of Public Health, Itabashi, Japan

**Keywords:** anxiety, COVID-19 pandemic, digital health literacy, fever, mHealth, parents

## Abstract

**Introduction:**

Fever in children is typically not an indication of severe illness; however, parental anxiety remains high. Recently, factors such as declining birth rates and the COVID-19 pandemic have influenced parental health awareness and behavior. This study aimed to evaluate the concerns and knowledge about fever among parents whose children visited the emergency department in 2017 and 2024, and to evaluate the changes over the 7 years and the association with the download of the smartphone app “Oshiete! Doctor,” which provides child healthcare information.

**Methods:**

These repeated cross-sectional surveys were conducted at the Holiday Pediatric Medical Center in Saku in 2017 and 2024. A questionnaire was administered to caregivers of children aged ≤8 years to assess their anxiety about fever (brain damage, seizures, and dehydration) and knowledge (need for antibiotics for fever, body temperature as a criterion for consultation, and the criteria for attending daycare). The awareness and utilization of the application were also investigated. Data from 2017 and 2024 were compared using the chi-square and *t*-tests.

**Results:**

A comparison between the 2017 survey (*n* = 224) and the 2024 survey (*n* = 261) revealed a significant increase in concerns about brain damage and seizures in response to fever (+14.4% and +14.9%, respectively). In contrast, substantial improvements were observed in the recognition of the necessity of antibiotics for fever, the recognition of body temperature as a criterion for consultation, and the criteria for attending daycare. When participants were stratified according to the application download status, anxiety levels increased in both groups; however, the magnitude of increase was slightly smaller in the download group. Nevertheless, statistical significance was not confirmed in the multivariable analysis.

**Discussion:**

Over the past 7 years, an increase in parents’ knowledge of fever and an escalation in their anxiety were observed. However, the dissemination of the application alone did not demonstrate an effect strong enough to offset the overall increase in societal anxiety. Future studies may be able to more clearly verify a suppressive effect on this increase by using more refined exposure measurements based on behavioral indicators, such as actual frequency of use, including by expanding the sample size.

## Introduction

Although fever in children rarely indicates severe illness, parental anxiety about fever is high (1). Parents often consulted a doctor when their child had a fever (2). Approximately two-thirds of parents typically visit a medical facility within the first 12 h after their child develops a fever (3). This strong anxiety about fever has been widely known since it was defined as “fever phobia” by Schmitt et al. in the 1980s (4). Fever phobia is characterized by parental anxiety and exaggerated, unrealistic misconceptions about fever, including concerns about brain damage, impaired consciousness, and hearing loss (5). Despite years of education, many parents still have misconceptions about fever.

In recent years, following the declining birth rates and the COVID-19 pandemic, parental awareness of health has changed. During the COVID-19 pandemic, a significant amount of medical information was disseminated; however, this did not necessarily result in effective health behaviors. Ishikawa et al. reported that health literacy declined during the pandemic (6). Given this context, awareness and behaviors associated with fever may also have changed. Kupcova et al. reported that anxiety worsened during the pandemic, particularly among women and young adults (7). During the pandemic, many public health institutions recommended treating fever with acetaminophen or ibuprofen (8). However, this may have resulted in a heightened sensitivity to fever and exacerbated anxiety related to fever. However, to our knowledge, no previous studies have examined the changes in fever-related anxiety or the behavior of parents before and after the pandemic.

To reduce parental anxiety, it is necessary to provide accurate knowledge and guidance on fever management. Parents seek more accessible and consistent information regarding fever management (9). Providing parents with appropriate health information about their children can increase their knowledge and decrease anxiety. Previous randomized controlled trials have reported that educational interventions, such as informational leaflets, significantly improved parents’ knowledge regarding fever, including its definition (temperature above 38 °C) and appropriate management strategies (10). Furthermore, a Randomized control trial showed that video tutorials guided managing common childhood symptoms and deciding when to seek medical help, significantly and safely increasing parents’ self-efficacy when used by people calling out-of-hour medical helplines (11). Recently, research on digital health has also been conducted. For example, FeverApp, an app that provides fever management protocols for parents in Germany, has been introduced. Beerenbrock et al. emphasize that structured guidance based on this app has the potential to reduce parents’ concerns about fever (12). Conversely, there is limited evidence on evaluating the effectiveness of such apps.

In 2015, a parent education initiative, “Oshiete! Doctor” (“Teach Me, Doctor”), was launched in Saku City, Nagano Prefecture, and a mobile application was developed to provide health information aimed at reducing parents’ anxiety. The application was designed to be free of charge and user-friendly, with a simple interface to facilitate ease of use and provide reassurance to parents during emergencies.

In 2022, this study surveyed to investigate the association between application use and parental health literacy (13). This study suggests that if parents have lower health literacy, they are less likely to use the app and may not be able to obtain sufficient benefits. Many questions remain regarding how providing information through applications can help reduce parental anxiety.

This study aimed to evaluate the fever-related anxiety and knowledge among parents of infants and young children who visited the emergency department with fever as the primary complaint in 2024, and to analyze the changes over the 7 year period by comparing the data with that from a similar survey conducted in 2017. Furthermore, this study evaluated the association between fever-related anxiety and behaviors and the download of mobile applications.

## Materials and methods

### Study participants and data collection

These repeated cross-sectional surveys were conducted at the Holiday Pediatric Medical Center in Saku City, Nagano Prefecture, in 2017 and 2023. The center is the only medical institution responsible for morning holiday outpatient services (no ambulances accepted) in the Saku region, which has a target population of 230,000. The survey period was from October 1, 2017, to January 30, 2018, and from December 20, 2024, to March 31, 2024. The study targeted guardians of children aged ≤8 years who visited the center during these periods, with 229 participants in the first period and 425 in the second period. The parents received an explanation of the survey from the nurses and participated in the survey with their consent. Participants who selected “agree to participate” in the questionnaire were regarded as having consented to the study. The questionnaire was collected by the nursing staff after consultation (Supplementary File S1). Respondents were excluded if they were not a child’s mother or father, did not own a smartphone, or visited the center more than once during the study period. The total number of respondents in the 2017 survey was 224 (224/229; response rate 98%). All of these respondents were individuals who had visited the medical facility because their child presented with fever symptoms. In the 2024 survey, the number of respondents was 386 (386/425; response rate 91%). Among these, 261 respondents who visited the medical facility due to their child’s fever symptoms were included as the study participants. This study was approved by the Ethics Review Committee of the Saku Central Hospital (Approval Number: R202310-05).

### Overview of the application

The “Oshiete! Doctor” application was launched in 2016 and is offered free of charge, with support from Saku City, to improve guardians’ childcare skills. The application provides information on seven topics: guidelines for seeking medical care for sick children, explanations of childhood illnesses, vaccination information, details of childcare support organizations, and disaster preparedness for children (See Supplementary File S2). This app has a section on fever management, which provides guidelines on how to determine whether to seek medical consultation and knowledge about home care for fever.

### Survey questions

#### Concerns about fever

Concerns about fever were assessed by asking whether participants agreed with the following three statements based on previous studies (2) (14): “Brain damage may occur,” “Convulsions may occur,” and “Dehydration may occur.” Responses were categorized as “concerned” if participants selected “strongly agree” or “somewhat agree” and as “not concerned” if they selected “somewhat disagree” or “strongly disagree.”

#### Knowledge about fever

Knowledge about fever was assessed using the following statement: “Antibiotics are necessary for the treatment.” “Body temperature serves as a criterion for seeking medical attention,” and “If there was no fever the previous day and the child is afebrile in the morning, they can attend school.” Responses regarding antibiotic necessity were categorized as “Necessary” if participants selected “Strongly agree” or “Somewhat agree” and as “Not necessary” if they selected “Somewhat disagree” or “Strongly disagree.” Responses regarding body temperature as a criterion for seeking medical attention were categorized as “Serves as a criterion” or “Does not serve as a criterion.” Responses regarding school attendance were classified as “Can attend school” or “Cannot attend school” after excluding the “Cannot decide” response. These questions were selected based on the following clinical facts: “Most cases of fever in children are viral infections and do not require antibiotics” (15); “The judgment to consult a doctor for a child should be based on an evaluation of the child’s overall condition and cannot be made based on body temperature alone” (16); and “It is not uncommon for children to have no fever in the morning and then develop a fever in the afternoon” (17). Although a scale to assess parents’ fever management practices has been developed overseas (18), it had not been validated in Japanese at the time of this study. Therefore, instead of that scale, this study employed these three questions commonly used to advise patients with fever in pediatric clinical practice.

#### Recognition and download of the “Oshiete! Doctor” application

The participants were asked about their recognition and download of the application. In the present study, only the download status was available; therefore, it was used as a surrogate measure for actual app use. Responses were categorized as “not downloaded” if they selected “do not know the application” or “know the application but have not downloaded it,” and as “downloaded” if they selected “have downloaded it.”

#### Socio-demographic data

The analysis included the respondent’s relationship with the child, age group, the child’s age and sex, and birth order. Respondent-child relationships were categorized as either “Father” or “Mother.” Age groups were classified into four categories: 20s, 30s, 40s, 50s, or older. Birth order was categorized as either “first child” or “second child and later.”

#### Data analysis and statistics

Continuous variables are summarized using descriptive statistics (mean and standard deviation or median and quartiles), and categorical variables are expressed as frequencies and percentages. Socio-demographic characteristics, application recognition and usage, knowledge and attitudes toward fever, and related behaviors were compared between the 2017 and 2024 surveys using chi-square or *t*-tests. This study also constructed a multivariable logistic regression model in which the survey year (2017 vs. 2024), application download status (no vs. yes), and their interaction (year × app) were the main explanatory variables, adjusted for the child’s age, birth order, and the parent’s age group. With regard to the handling of missing values, a complete-case analysis was conducted.

Data analysis was performed using the Stata 17 software (StataCorp LP, College Station, Texas, United States).

## Results

In the 2017 survey, 224 out of 229 parents responded to the questionnaire (response rate of 98%). All of their children presented with fever. In the 2024 survey, 386 of the 425 parents responded (response rate of 91%). Of these respondents, 261 were parents of children with fever. Table 1 presents the respondents’ background characteristics. The proportion of fathers among respondents showed a slight increase from 19.6% in 2017 to 24.1% in 2024; however, this difference was not statistically significant. The most common age group among parents in both surveys was the ≥30s. However, the proportion of parents in this age group decreased from 66.1% in 2017 to 51% in 2024, whereas the proportion in other age groups increased. No significant differences were observed in the age and sex of the children or the proportion of first-born children between 2017 and 2024.

**Table 1:** Background characteristics of parents and children and awareness and utilization of the application.

Characteristics	2017 *n* = 224		2024 *n* = 261		*p*-value*
Relationship					0.234
Father	44	19.6%	63	24.1%	
Mother	180	80.4%	198	75.9%	
Age group of parents					**0.026**
20s	25	11.2%	39	15.4%	
30s	148	66.1%	133	52.6%	
40s	49	21.9%	76	30.0%	
50s and older	2	0.9%	5	2.0%	
Age of child (years)	3.7 ± 2.6		4.0 ± 2.5		
Sex of child					0.243
Male	123	54.9%	132	50.6%	
Female	97	43.3%	129	49.4%	
Birth order					0.827
First	104	49.3%	126	48.3%	
Second or later	107	50.7%	135	51.7%	
Recognition and download of the App					**<0.001**
Do not know the app	107	47.8%	88	34.0%	
Know the App, but have not downloaded it	45	20.1%	108	41.5%	
Have downloaded the App	72	32.1%	64	25.0%	

*P-values were generated using a *t*-test or χ^2^ test. *P* values indicating statistically significant differences (*p* < 0.05) were shown in bold.

Regarding awareness of the application, the proportion of parents who knew the application increased from 52% in 2017 to 66.5% in 2024. However, the proportion of parents who reported downloading the application decreased from 32% in 2017 to 25% in 2024.

Table 2 presents a stratified comparison of concerns regarding fever, categorized by the download status of the application between 2017 and 2024. Overall, concerns about brain damage and seizures increased in 2024 compared to those in 2017. Particularly, the proportion of parents who expressed concerns about brain damage and seizures was significantly higher in the non-download group between 2017 and 2024. However, in the multivariable logistic regression analyses regarding concerns about brain damage, seizures, and dehydration (not shown in the table), none of the year × application interactions were statistically significant (*p* = 0.956, *p* = 0.306, and *p* = 0.903, respectively). In other words, no evidence was obtained to suggest that the magnitude of change from 2017 to 2024 differed according to application use. The child’s age showed an independent positive association with anxiety about brain damage and seizures (Adjusted odds ratio [aOR] = 1.1 and 1.2, respectively). No clear associations were observed with birth order or the parents’ age group.

**Table 2:** Comparison of concerns related to fever by application download (2017 and 2024).

Concerns related to fever	Group	2017	2024	*p*-value*
Brain damage	Total	115/217 (53.0%)	174/259 (67.2%)	0.001
	Non-download group	79/150 (52.7%)	131/194 (67.5%)	**0.005**
	Download group	36/67 (53.7%)	43/64 (67.2%)	0.116
Seizure	Total	142/216 (65.7%)	209/259 (80.7%)	<0.001
	Non-download group	95/150 (63.3%)	158/194 (81.4%)	**<0.001**
	Download group	47/66 (71.2%)	50/64 (78.1%)	0.365
Dehydration	Total	191/216 (88.4%)	227/261 (87.0%)	0.71
	Non-download group	133/150 (88.7%)	173/196 (88.3%)	0.908
	Download group	58/66 (87.9%)	54/64 (84.4%)	0.563

^*^P-values were generated using a *t*-test or χ^2^ test. *P* values indicating statistically significant differences (*p* < 0.05) were shown in bold.

Table 3 presents a stratified comparison of parental knowledge regarding fever, categorized by application download status, between 2017 and 2024. The proportion of respondents who believed that antibiotics were necessary for fever, those who considered body temperature to be a criterion for seeking medical care, and those who allowed children to attend day care if they were afebrile on the day of attendance despite having a fever the previous day all demonstrated a substantial decrease. This trend was observed regardless of the download status of the application. Similarly, in the multivariable logistic regression analysis, knowledge regarding fever improved from 2017 to 2024 in all items; however, none of the year × application interactions were statistically significant (*p* = 0.238, *p* = 0.165, *p* = 0.998), and no evidence was obtained that “the degree of change from 2017 to 2024 differed according to application use.

**Table 3:** Comparison of knowledge related to fever by application usage (2017 and 2024).

Knowledge related to fever	Group	2017	2024	*p*-value*
Antibiotics were necessary for the fever	Total	117/217 (53.9%)	93/258 (36.1%)	**<0.001**
	Non-download group	89/151 (58.9%)	73/194 (37.6%)	**<0.001**
	Download group	28/66 (42.4%)	20/63 (31.8%)	0.21
Consider body temperature as a criterion for seeking medical care	Total	191/215 (88.8%)	99/229 (43.2%)	**<0.001**
	Non-download group	134/149 (89.9%)	71/173 (41.0%)	**<0.001**
	Download group	57/66 (86.4%)	28/56 (50.0%)	**<0.001**
If the fever is gone in the morning, the child can attend school.	Total	40/183 (21.9%)	11/254 (4.3%)	**<0.001**
	Non-download group	28/124 (22.6%)	9/192 (4.7%)	**<0.001**
	Download group	12/59 (20.3%)	2/62 (3.2%)	**0.003**

*P-values were generated using a *t*-test or χ^2^ test. *P* values indicating statistically significant differences (*p* < 0.05) were shown in bold.

## Discussion

This study aimed to compare changes in anxiety and knowledge about fever between 2017 and 2024 among parents of children aged ≤8 years who visited a holiday medical center with a chief complaint of fever, and to assess the association between these factors and the download of the Japanese parent-oriented app “Oshiete! Doctor.”

The importance of health literacy has been emphasized during the pandemic (19). Particularly, digital health literacy has been reported to influence individuals’ ability to access, understand, and evaluate information and services that support healthy behaviors during a pandemic (20). In the present study, the proportion of parents who had downloaded the application in 2024 among those visiting a holiday medical care center was 25%. In contrast, a 2023 study targeting parents who attended infant health checkups in the same region found that 43.4% of them had downloaded and utilized the application (13). In Japan, infant health checkups are nearly universal, with a very high participation rate. Therefore, this result reflects the overall utilization rate of the app by parents in this region. Consequently, it is suggested that parents who seek emergency outpatient care are less likely to download the application than the general parent population. It is also possible that parents who downloaded the application may have avoided emergency outpatient visits.

Furthermore, it was found that among parents visiting holiday medical care centers, awareness of the application increased between 2017 and 2024. However, the proportion of those who downloaded the application decreased. After the COVID-19 pandemic, parents who actively used the application may have become more inclined to refrain from emergency outpatient visits. The comparison of these survey data before and after the pandemic suggests an association between app downloads and reduced emergency outpatient visits, indicating that digital health literacy may be associated with preventive behavior during the pandemic.

The present study demonstrated a substantial decline in 2024 compared to 2017 in the proportion of respondents who acknowledged fever as a valid indication for seeking medical intervention, including those who regarded fever as a suitable reason to skip school if it had subsided the previous day. An elevated body temperature does not necessarily indicate severe infectious disease; therefore, it should not be used as a criterion for seeking medical care (21). Furthermore, owing to the influence of the circadian rhythm, body temperature tends to be higher in the afternoon than in the morning (22). Therefore, if a fever is present on a given day, there is a possibility of recurrence in the afternoon, even if the fever subsides in the morning. This underscores the importance of rest and the necessity of refraining from attending school. Consequently, these changes in knowledge are considered favorable, suggesting that parental understanding of fever has improved over the past 7 years. This may be due to the increase in public messages during COVID-19, such as appropriate fever management and the limited role of antibiotics, which may have influenced parents’ knowledge.

With respect to the proportion of caregivers who believed that antibiotics were necessary in the presence of fever, in 2017 the figures were 89/151 (non-download group, 58.9%) versus 28/66 (download group, 42.2%), indicating that the download group provided fewer incorrect responses (*p* = 0.025), suggesting an association between app download and higher health literacy. However, in 2024, the proportion of incorrect responses decreased in both groups, with 73/194 (37.6%) in the non-download group and 20/63 (31.8%) in the download group, and the difference between the groups had narrowed. In Japan, nationwide campaigns promoting the appropriate use of antibiotics have been implemented to address antimicrobial resistance as the Action Plan for Countermeasures against Antimicrobial Resistance (AMR) since 2016. Initiatives, such as monthly antimicrobial resistance awareness, have been implemented to promote public education on the proper use of antibiotics. Furthermore, educational programs for healthcare professionals have been implemented to support the appropriate use of antibiotics. Some reports indicated a decrease in antibiotic consumption in Japan following the launch of these campaigns (23). This study’s findings may also suggest the effectiveness of the nationwide campaign promoting the appropriate use of antibiotics.

Conversely, the proportion of individuals expressing concerns about brain damage and seizures related to fever increased over the past 7 years. This may be attributed to the influence of media coverage related to the COVID-19 pandemic and the heightened uncertainty, which increased parental vigilance and anxiety. Previous studies have also reported that health-related anxiety worsens during pandemics (7).

When participants were stratified according to the presence or absence of application download, anxiety levels increased in both groups; however, the magnitude of increase was slightly smaller in the group that had downloaded the application, suggesting a potential mitigating effect on the rise in anxiety. We have previously reported that individuals unfamiliar with the application exhibited comparatively lower health literacy (13). Parents with lower digital health literacy may have been less likely to download the application and, as a result, may have demonstrated higher levels of anxiety. However, in multivariable analysis, the statistical significance of the interaction was not confirmed, which may be attributable to insufficient statistical power or to the fact that the exposure was measured as “download or not,” rather than capturing the actual intensity of use. This may have introduced non-differential exposure misclassification (download ≠ actual use). With more refined exposure measurements based on behavioral indicators such as actual frequency of use and type of content accessed, including an expanded sample size, it may be possible to more clearly verify the potential attenuating effect on the increase in anxiety.

If the use of the smartphone application is associated with better knowledge and lower unnecessary fear of fever, this could lead to more appropriate home management of fevers and possibly fewer unnecessary emergency department visits or calls to pediatricians. This aligns with public health goals of focusing medical attention where it is truly needed and empowering parents to care for mild illnesses at home. Therefore, the use of such apps and improved health literacy may also lead to a reduction in the burden on the healthcare system.

However, these studies are repeated cross-sectional surveys, and the inference of a causal relationship between application download and the reduction of anxiety is limited; these mechanisms remain at the hypothetical stage. Further research is needed to demonstrate these hypotheses.

This study has several limitations. First, the study period was limited to the winter season from December to March. Although numerous diseases cause fevers, pediatric illnesses often exhibit seasonality. Thus, conducting the study exclusively during winter may have influenced the results. However, winter is the peak season for emergency department overcrowding due to influenza outbreaks and other factors. This study aimed to explore measures to alleviate the burden of emergency services. Therefore, evaluating this specific period was of utmost importance, and seasonal selection was deemed appropriate.

Second, the participants included in the studies conducted in 2017 and 2024 were not from the same cohort. Consequently, individual-level changes could not be assessed. However, the participants were parents of children aged ≤8 years or younger who sought medical attention at the same holiday medical center during winter because fever was the primary complaint. Given the similarities in their backgrounds, valid comparisons could be made at the group level.

Third, although the present study examined the proportion of parents who downloaded the mobile application, it did not evaluate the extent to which they utilized it after downloading. Therefore, differences were observed by download status, but the extent of app use was not measured. App download coarsely measures the true exposure (app use), and because participants who downloaded but did not use the app were included in the use group. If reanalysis were to be conducted using app use, the effect estimate would likely appear stronger, and we believe this would affect the results of multivariable analysis.

Fourth, since there is only one holiday medical center for children in this area, this study is considered to be representative of the emergency consultation behavior of parents in this area. However, since this study was conducted in a single region, region-specific factors may influence the results, which limits the generalizability of the findings to other regions.

Fifth, in the 2017 survey, the questionnaire was administered to parents whose children visited the medical facility with fever, whereas in 2024, the questionnaire was administered to all parents whose children visited the medical facility, and from among them, those with children presenting with fever were identified. Because the recruitment methods in the 2 years were not completely identical, there is a possibility that the comparison may have been rendered less precise. However, we consider that this methodological difference was minimal and likely had little actual impact on the findings.

Furthermore, as this study is based on self-reported data, it may be subject to response bias. Conversely, respondents may have overestimated their knowledge or underreported their concerns due to social desirability, which may have affected the results.

Finally, this study comprises two repeated cross-sectional surveys. Therefore, it is necessary to consider the insufficient control of potential confounding factors and the possibility of reverse causation between application use and anxiety, and it should be noted that causal relationships cannot be inferred from this study.

Parental knowledge of fever may have improved following the COVID-19 pandemic. In particular, there is a suggestion that awareness of refraining from antibiotic use has increased, indicating that campaigns promoting the appropriate use of antibiotics may have been effective. In contrast, anxiety related to fever tended to worsen, with this trend being more pronounced among parents who had not downloaded the application. In the future, longitudinal research targeting multiple regions that directly measures broad contextual factors such as application use, parental health literacy, and.

pandemic-related stress is needed to study the causal relationship between measures to improve digital health literacy and the reduction of parents’ concerns.

## Data Availability

The raw data supporting the conclusions of this article will be made available by the authors, without undue reservation.
