# Supplementary material for: Changes in parental knowledge and concerns regarding pediatric fever from 2017 to 2024: repeated cross-sectional surveys on the association of a smartphone application
Source: Front Public Health. 2026 Jan 6;13:1619134. doi: 10.3389/fpubh.2025.1619134 (PMC12816234; doi:10.3389/fpubh.2025.1619134)
Supplement: Supplementary file 1 [file Supplementary_file_1.pdf]

Questionnaire for Parents/Guardians of Children Who Visited for Fever

1. Please tell us your child's age, sex, number of siblings, and birth order.
   Year: (   ) years old   Sex: (□ Male □ Female)

   Number of siblings: ____   Birth order: the (   )th child

2. Who is filling out this form?
   □ Father □ Mother □ Grandfather □ Grandmother □ Other

3. Please tell us the age of the respondent.
   □ Teens □ 20s □ 30s □ 40s □ 50s

4. What was the temperature at the time of the visit? ____ ℃

5. Other than fever, are there any symptoms?
   □ Runny nose □ Cough □ Diarrhea □ Vomiting □ Rash □ Other (____)

6. When your child has a fever...

   1) Brain damage might occur.
   □ Strongly think so □ Think so □ Do not really think so □ Do not think so

   2) They might have a seizure.
   □ Strongly think so □ Think so □ Do not really think so □ Do not think so

   3) They might become dehydrated.
   □ Strongly think so □ Think so □ Do not really think so □ Do not think so

   4) I think antibiotics are necessary.

   □ Strongly think so □ Think so □ Do not really think so □ Do not think so

7. Even in a child who is otherwise well, from what temperature and above do you think a fever requires an urgent visit?
   □ 37.0°C □ 37.5°C □ 38.0°C □ 38.5°C □ 39.0°C □ 39.5°C □ 40.0°C
   □ If there is only fever and the child is well, an urgent visit is not necessary

8. If there was a fever last night but this morning the fever is gone and your child is well and in a good mood, what would you do?

□ Watch at home for one more day □ Have them attend daycare/school

9. Are you aware of the Saku Medical Association “Oshiete Doctor!” app?

□ Do not know it □ Know it but have not downloaded it □ Have downloaded it
