# Supplementary figures and images for: Changes in parental knowledge and concerns regarding pediatric fever from 2017 to 2024: repeated cross-sectional surveys on the association of a smartphone application
Source: Front Public Health. 2026 Jan 6;13:1619134. doi: 10.3389/fpubh.2025.1619134 (PMC12816234; doi:10.3389/fpubh.2025.1619134)

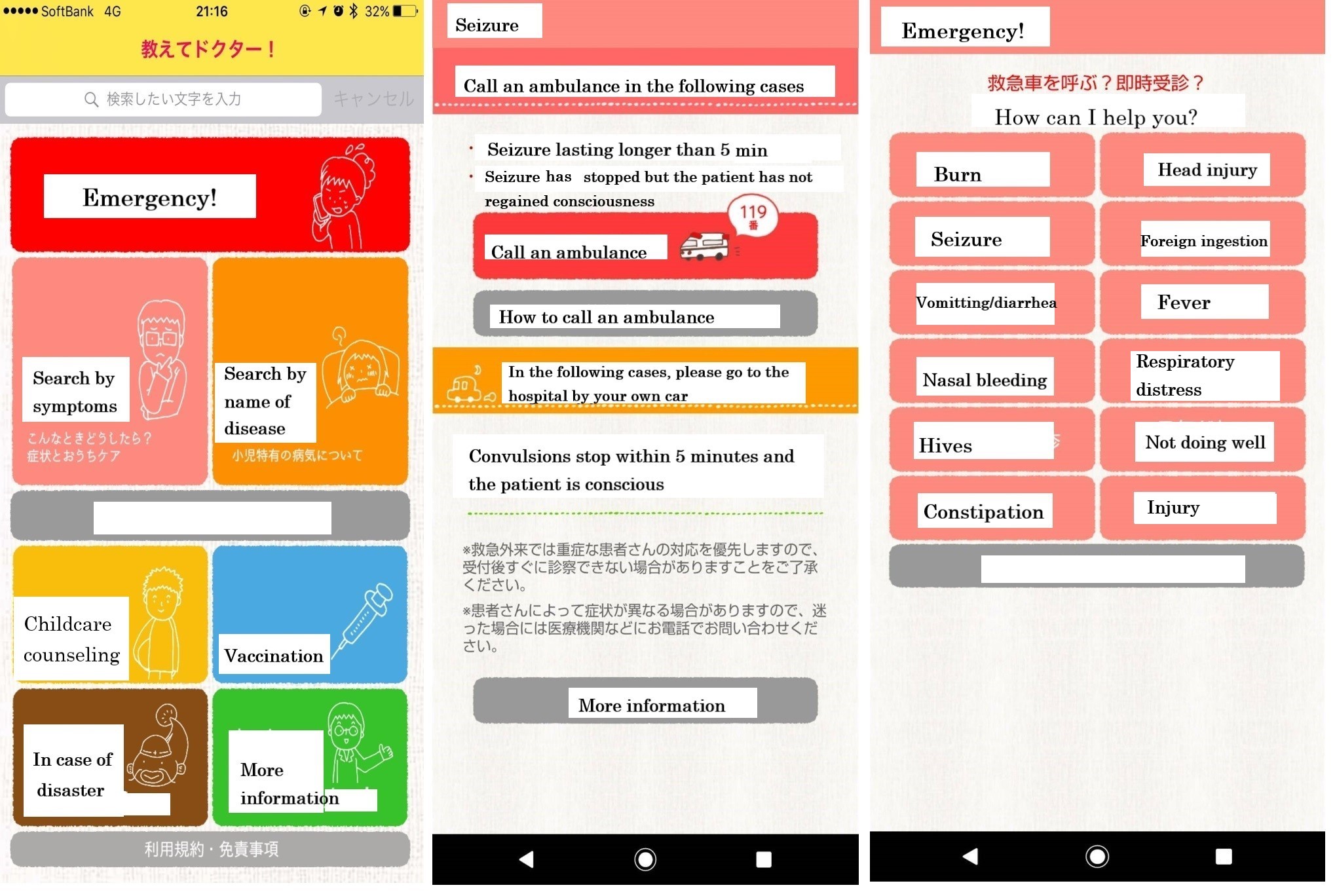

Supplement: Supplementary file 2 [file Supplementary_file_2.jpeg]
